# Protection of diabetes in aortic abdominal aneurysm: Are antidiabetics the real effectors?

**DOI:** 10.3389/fcvm.2023.1112430

**Published:** 2023-03-23

**Authors:** Belén Picatoste, Isabel Cerro-Pardo, Luis M. Blanco-Colio, Jose L. Martín-Ventura

**Affiliations:** ^1^ Laboratory of Vascular Pathology, IIS-Fundación Jiménez Díaz, Madrid, Spain; ^2^ Biomedicine Department, Alfonso X El Sabio University, Madrid, Spain; ^3^ CIBERCV, Madrid, Spain; ^4^ Medicine Department, Autonoma University of Madrid, Madrid, Spain

**Keywords:** abdominal aortic aneursym, diabet mellitus, insulin resistance, obesity, antidiabetic drugs

## Abstract

Aortic aneurysms, including abdominal aortic aneurysms (AAAs), is the second most prevalent aortic disease and represents an important cause of death worldwide. AAA is a permanent dilation of the aorta on its infrarenal portion, pathologically associated with oxidative stress, proteolysis, vascular smooth muscle cell loss, immune-inflammation, and extracellular matrix remodeling and degradation. Most epidemiological studies have shown a potential protective role of diabetes mellitus (DM) on the prevalence and incidence of AAA. The effect of DM on AAA might be explained mainly by two factors: hyperglycemia [or other DM-related factors such as insulin resistance (IR)] and/or by the effect of prescribed DM drugs, which may have a direct or indirect effect on the formation and progression of AAAs. However, recent studies further support that the protective role of DM in AAA may be attributable to antidiabetic therapies (i.e.: metformin or SGLT-2 inhibitors). This review summarizes current literature on the relationship between DM and the incidence, progression, and rupture of AAAs, and discusses the potential cellular and molecular pathways that may be involved in its vascular effects. Besides, we provide a summary of current antidiabetic therapies which use could be beneficial for AAA.

## 1. AAA and DM

**Figure 1:**
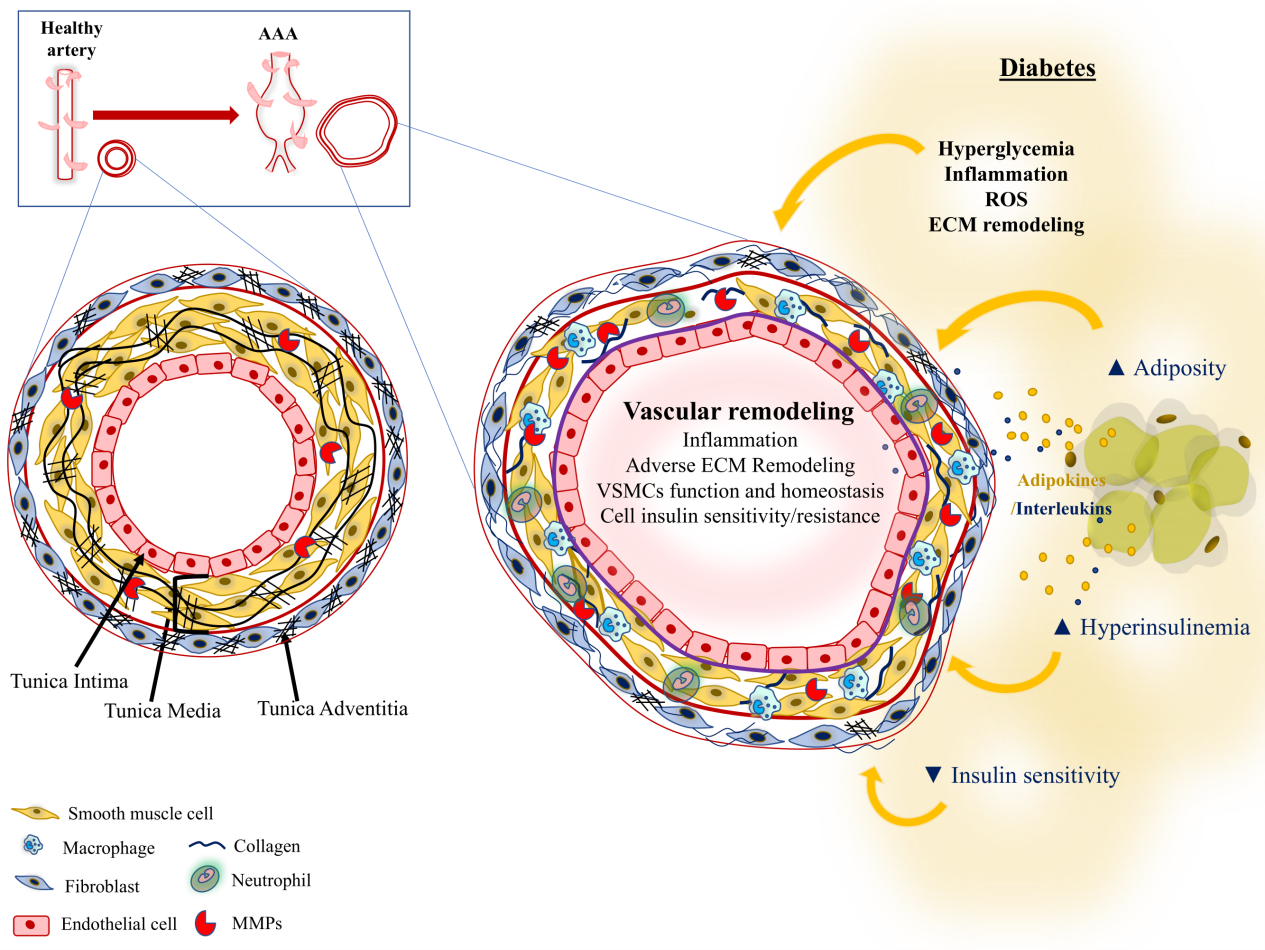
Pathological processes in type 1 and/or type 2 diabetes potentially affecting AAA progression.

Aortic aneurysms, including abdominal aortic aneurysms (AAA), is the second most prevalent aortic disease following atherosclerosis, affecting approximately 1%–2% of all 65-year-old men and therefore, representing an important cause of death worldwide (1). AAA is a local dilatation of the abdominal aorta larger than 3 cm or exceeding by 50 per cent the normal aortic diameter (2) and is pathologically associated with oxidative stress, proteolysis, extracellular matrix (ECM) remodeling and degradation, vascular smooth muscle cell (VSMC) loss and immune-inflammatory responses (3, 4) (Figure 1). A significant change in the management of patients with AAA has occurred during the last 15–20 years, which has improved mortality rates after surgery treatment (5). In addition, screening programs and changes in sociodemographic and social trends, is helping reducing rupture incidence and mortality rates (1, 6–8). AAAs are often asymptomatic until rupture and incidentally detected by ultrasound, x-rays, or CT scans when patients are examined for a non-related reason. Several risk factors for AAA have been identified including aging, male sex, hypertension, dyslipidemia, and smoking (9). Prior studies have failed to identify specific treatments for AAA and randomized clinical trials have supported surgery (open or endovascular surgery) only in patients with large (aortic diameter > 5–5.5 cm) or symptomatic AAAs (2, 10).

Diabetes Mellitus (DM), a disease which is characterized by chronic hyperglycemia, induces nephropathy and microvascular disease, and represents a major risk factor for cardiovascular disease (CVD) (80 percent of deaths among those with DM) (11, 12). Type 1 DM (T1DM) and Type 2 DM (T2DM) have abnormally elevated blood glucose levels as main characteristic of the disease but differ in many other aspects. While T1DM is primarily the result of autoimmune destruction of pancreatic beta cells, T2DM is characterized by the loss of insulin sensitivity, deficiency in insulin secretion and is frequently linked to obesity (13). Although DM is one of the main risk factors for CVD, most epidemiological studies have described its potential protective role on the prevalence and incidence of AAA (14–18).

For non-post-operative data, most studies suggest the protective role of DM in AAA (14). Meta-analysis of prospective studies demonstrate that people with DM have lower prevalence of AAA (19–22) and that people with DM and small AAAs (aortic diameter between 3 and 5 cm) have slower AAA progression (22–24). The VIVA trial showed an inverse relationship between HbA1c blood levels and AAA growth rate (24). A recent study in 250 subjects showed that patients with DM have more than a 35 percent reduction in the median AAA growth rates despite having more severe concomitant vascular comorbidities (25). A retrospective study on the relationship between DM and AAA rupture showed that patients with DM and AAA are significantly less likely to present AAA rupture or to die from AAA rupture when compared to nondiabetic patients with AAA (26). However, the authors of this study suggested that the protective effect on AAA could be due to DM itself or to the pharmacological treatments of DM. According to these results, a meta-analysis of 9 studies of AAA rupture and 2 studies of non-rupture symptomatic AAA demonstrated that DM was associated with lower prevalence of AAA rupture (18). Although DM and/or antidiabetic drugs seems to exert a protective effect on the vascular wall, their effect on the post-operative cardiovascular outcomes after open surgery in patients undergoing AAA repair is more controversial. In this sense, a recent nationwide study in France identified T1DM as a risk factor of post-operative mortality in patients undergoing AAA repair (27). Some reports show increased post-operative mortality in patients with DM compared to controls (22, 28). However, some others report lower mortality (19). The adjusted analysis in a Swedish nationwide observational study showed that patients with DM have significantly lower risk of total and cardiovascular mortality after acute aortic repair, whereas rates of cardiovascular events, acute myocardial infarction and stroke did not differ between groups (29). There are also some studies which show no differences in post-operative mortality between patients with and without DM (17, 30–32). One of those studies showed that patients with DM had higher rates of acute myocardial infarction and major adverse cardiovascular events after elective open AAA repair than those without DM, despite not finding differences in post-operative mortality (31).

The research on the protective role of DM in AAA has been mainly tested on animal models. To our knowledge, these studies have been mainly performed in models of T1DM (15). Streptozotocin-induced T1DM shows a protective effect in AAA induced by either by elastase infusion in the abdominal aorta of C57BL/6 mice or by Angiotensin II (Ang II) infusion in apolipoprotein E knock-out (ApoE^−/−^) mice (33, 34). Dr. Zhonglin Chai's group demonstrated that cell division autoantigen 1 (CDA1) is upregulated in DM and enhances TGF-β signaling, including the vasculature (35). In this study, the authors demonstrated that deletion of CDA1 in mice with DM decreased TGF-β signaling and reduced ECM accumulation, which would revert the protective effect of DM and then, contribute to aneurysm formation (36, 37). Importantly, stimulation of TGF-β signaling prevents AAA (38, 39) and blockade of TGF-β accelerates AAA development (39, 40). Thus, the protective effect of CDA1 could be explained by its effect on TGF-β signaling. A recent study in mice has suggested the potential contribution of dysregulated prolyl hydroxylase domain (PHD) containing proteins to DM mediated AAA suppression (41). The authors proposed that AAA attenuation in the setting of DM was derived from enhanced aneurysmal angiogenesis as a consequence of dysregulated PHD activity. On the other hand, mice models of T2DM present hyperglycemia and insulin resistance (IR), often associated with obesity (42), which fairly recapitulates human disease. However, to our knowledge, although Tanaka et al. studied the effect of T2DM on carotid aneurysm (by using KK-Ay mice), no mouse experiments have been performed to study the effect of T2DM on AAA, which implies a lack of information on the potential mechanisms by which DM would exert protective effect on AAA.

In spite of the commented clinical and experimental evidence suggesting a protective role of DM in AAA, a Mendelian randomized analysis recently demonstrated that lifelong genetic predisposition to T2DM does not protect against AAA (43). Moreover, human diabetic arteries have intrinsic properties potentially related to AAA development, such as increased stiffness (44), endothelial dysfunction (45, 46) and calcifications (47). In this respect, DM due to the combination of hyperglycemia (T1 and T2DM) and obesity and IR (T2DM) could lead to pathological vascular remodeling through mechanisms including oxidative stress, inflammation and ECM degradation (Figure 1). In the next sections, we will show the discrepancy between some studies showing a protective role of DM in AAA with others supporting the potential contribution of DM-related factors to pathological mechanisms involved in AAA development.

### 1.1. Hyperglycemia and AAA

Hyperglycemia, a common risk factor for T1DM and T2DM, drives inflammation and reactive oxygen species (ROS) production, impairing endothelial cell and VSMC function (48–52). In addition, hyperglycemia accelerates senescence in endothelial cells (52, 53), VSMCs (54, 55), endothelial progenitor cells (56) and mesenchymal stem cells (57), which contributes to vascular dysfunction. Hyperglycemia induces changes in VSMC responses to vascular injury (55), which have been described to be mediated, at least in part, by β3 integrin signaling (55) and by suppression of insulin receptor substrate-1-mediated p53/KLF4 complex stabilization (58). DM and chronic hyperglycemia drive advanced glycation end products (AGE) production, activates glucose autooxidation and inhibits the production of antioxidant agents, all together influencing the activation of circulating and resident cells of the vascular wall. Thus, diabetes would be expected to be a risk factor for the development of AAA. However, hyperglycemia has been shown to stabilize the collagen network by generating a thicker aortic wall which exert a protective effect on the wall stress in the abdominal aorta of patients with DM (23, 59). In this regard, accumulation of collagen IV is commonly observed in human diabetic arteries (60, 61), while its deficiency is associated with AAA development (62). Moreover, circulating collagen IV degradation fragments correlated with AAA progression in the VIVA cohort (62). Furthermore, Golledge et al. suggested that the progression of AAA is slower in patients with diabetes through changes in monocyte-ECM interactions (23). Moreover, an study in AAA biopsies obtained from diabetic and nondiabetic patients suggested that cross-linking AGEs play a protective role in AAA progression in diabetic patients (63). Besides, Miyama et al. showed that hyperglycemia reduces progression of AAA disease in two models of elastase and Ang II infusion in mice (34). This study demonstrated that insulin treatment attenuates this protective effect. Identifying the mechanisms by which hyperglycemia exert a protective role against AAA formation and progression would contribute to the development of novel clinical therapies for AAA disease.

### 1.2. Obesity and AAA

Observational studies, interventional studies and post-hot analysis of clinical trials demonstrate that T2DM long-term glucose fluctuation is correlated with an increased risk of micro- and macro-vascular complications (64–71). However, it is conceivable that long-term variability of other DM-related risk factors (insulin levels, blood pressure, dyslipidemia, heart rate, body weight, and serum uric acid) may be involved in the development of AAA.

Obesity is a major health problem worldwide and is a major risk factor of the pathophysiology of T2DM and weight gain, promotes diabetes progression and is associated with worse glycemic control. Obesity is a risk factor for human AAA (72–74). The Physician's Health Study showed that, relative to men with body mass index (BMI) < 25, overweight (BMI = 25–30) and obese (BMI ≥ 30) men had 30%–70% higher risk of developing AAA (75). The same prospective analysis in the Physicians' Health Study showed that despite obesity was associated with a higher risk of AAA, the history of DM tended to associate with a lower risk of diagnosed AAA, particularly over longer follow-up. Increased adipose tissue mass has been shown to promote the two main defects of diabetes: IR and beta-cell dysfunction. A study on a Swedish population found that the risk of AAA was 30% higher in individuals with increased waist circumference (abdominal adiposity) (76). Moreover, patients with metabolic syndrome have a more than two fold increased risk for the development of T2DM, cardiovascular morbidity and mortality (77, 78). A recent study in 354 patients concluded that metabolic syndrome proportionally aggravates the progression of AAA (79). Interestingly, although the rupture rate was significantly higher and the survival rate significantly lower in patients with metabolic syndrome, the size of AAA was significantly smaller in patients with DM compared to patients with no DM (despite being metabolic syndrome more prevalent in the DM group) (79).

Regional differences in periaortic adipocytes and their differential ability to promote chemokine release, macrophage infiltration, and proinflammatory cytokine expression was related to enhanced AAA risk in obesity. The presence of adipocytes in the vascular wall is associated with AAA development and/or rupture (74). One of the main features in AAA pathogenesis is inflammation, which is often associated with the excess of PVAT. The amount of PVAT is higher in the aortas of patients with AAA (80). However, the molecular influence of PVAT on AAA has not been well described. Adipose tissue depots produce proinflammatory adipokines (leptin, TNFα and resistin) which have been suggested to have a role on AAA development. On the other hand, adiponectin, an adipokine which is positively involved in glucose and lipid systemic homeostasis, is decreased in the context of obesity (81). Interestingly, experimental AAA models have shown that adiponectin infusion reduces vascular inflammation, prevents vascular infiltration of macrophage and attenuates AAA development (82, 83). A recent systematic review and meta-analysis of animal and human observational studies, concluded that, although leptin and adiponectin upregulation do not affect AAA in animal models, studies in humans showed that resistin and leptin serum levels together with the amount of PVAT were higher in patients with AAA compared to control (84).

Besides autocrine and paracrine effect of fat tissue, adipocyte metabolism and molecular signaling might influence AAA development. It has been shown that genetic deletion of Ang II type 1a receptor in PVAT reduces AAA formation in ApoE^−/−^ mice (85). In 3T3-L1 adipocytes, Ang II inhibits insulin-stimulated IR and IRS-S tyrosine-phosphorylation, Akt activation, and glucose uptake (86). However, not only adipocytes but also other types of cells within the adipose tissue have been suggested to have a role on the progression of AAA. In this sense, macrophages within the adipose tissue are key in the inflammatory process of AAA due to their role on the degradation of the ECM (72). Diet-induced obesity as well as the leptin-deficient (ob/ob) genetic mouse model of obesity promotes macrophage infiltration in PVAT surrounding abdominal aortas and further increases Ang II-induced AAAs (72).

Although obesity is known to be one of the main risk factor of T2DM, the inverse correlation of DM with AAA could suggest a protective role of obesity against AAA. However, as mentioned before, adiposity has been implicated in other pathogenic mechanisms of relevance to AAA, suggesting that the relationship between obesity and AAA may be complex. Specific studies on the effect of DM with or without DM are needed to elucidate the contribution of both diseases on the pathophysiology of AAA.

### 1.3. Insulin resistance and AAA

Multiple studies have highlighted the association between DM and AAA. However, no conclusive studies have been done to elucidate the link between IR, one of the most important characteristics of T2DM, and the incidence and progression of AAA.

Systemic IR can cause vascular dysfunction and accelerate vascular disease in people with DM and metabolic syndrome (87–89). A clinical study shows an association of AAA diameter with IR (90). In this study, C-peptide, insulin concentrations and IR index (HOMA2 IR) were significantly higher in patients with AAA > 5 cm compared to those with AAA < 5 cm. IR might promote incidence and AAA progression by systemic factors (dyslipidemia, hyperglycemia, inflammation) as well as by the disruption of insulin signaling in endothelial cells, VSMCs and/or macrophages. Interestingly, insulin receptor-mediated signaling pathways in these cells are down-regulated in hyperinsulinemic environment (88, 91). In this sense, altered insulin signaling might be due to down regulation of insulin receptor signaling or by the excessive insulin receptor signaling (87). Studies in endothelial cells suggest that downregulation of insulin receptor may contribute to vascular remodeling in an insulin resistant environment (91, 92). A recent study focused on macrophages as potential responsible of the potential protective effect of DM on AAA showed that stimulation of macrophages with serum from patients with DM and AAA increase macrophage metabolism (based on differences in extracellular acidification and the expression of genes involved in glycolysis and lipid oxidation), which is accompanied by a shift towards an anti-inflammatory state (93). IR is accompanied by hyperinsulinemia and literature suggests that insulin modulates the inflammatory response in macrophages. In this regard, most of the studies show that insulin enhances inflammatory response and secretion of inflammatory cytokines (94, 95). However, there are also studies showing no effect on cytokine production by the macrophages in response to insulin, or even demonstrate the potential anti-inflammatory effect of insulin (96–98). Although there are several published studies on the mechanism of IR vascular remodeling, it remains unexplored whether endothelial, VSMC and macrophage IR affects the incidence and progression of AAA.

## 2. Oral antidiabetic drugs and AAA

**Figure 2:**
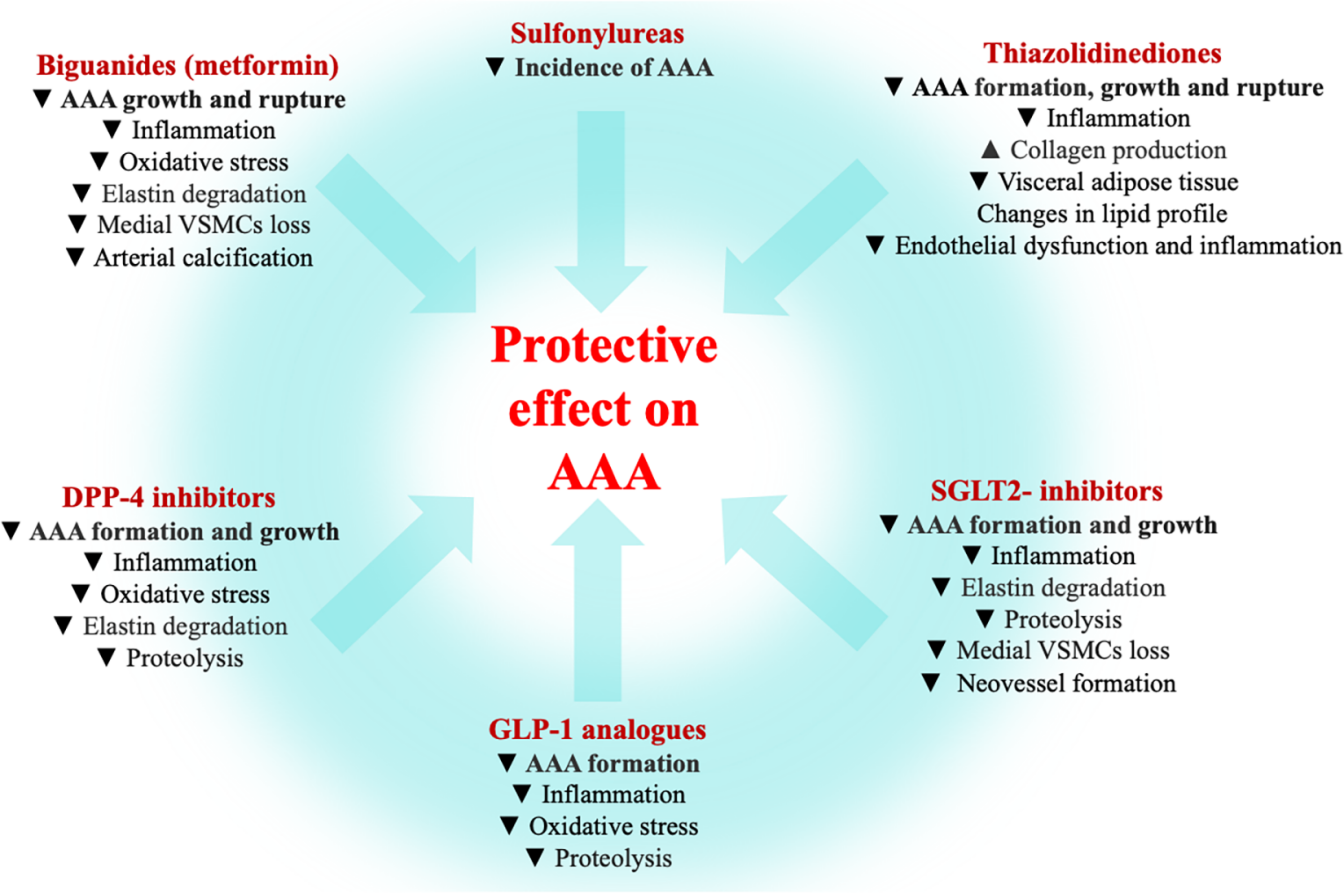
Antidiabetic agents and AAA. Summary of the mechanisms by which biguanides (103–124), sulfonylureas (131), thiazolidinediones (125–130), dipeptidyl peptidase inhibitors (DDP-4) inhibitors (149, 151–155, 158), glucagon-like peptide-1 (GLP-1) analogues (145–149, 156–158) and type-2 sodium-glucose co-transporter (SGLT2)-inhibitors (133–144) exert a protective effect on AAA (data from experimental and clinical studies).

Treatment of patients with DM includes lifestyle modifications (mainly healthy diet and exercise), together with a proper pharmacologic therapy (99–101) (Table 1). Drugs used to treat patients with T2DM have a variety of mechanisms by which they lower glucose levels (102), with different molecular targets and potential uses for other diseases. Recent available literature shows protective effects of some antidiabetic drugs on the incidence and progression of AAA (Figure 2).

**Table 1:** Antidiabetic drugs used in AAA patients.

Class of antidiabetic medication (route of administration)	Medicine class	Active ingredient
Oral	Sulfonylureas	Chlorpropamide, Tolazamide, Tolbutamide, Acetohexamide, Glipizide, Glyburide, Gliclazide, Glimepiride, Glibenclamide
	Meglitinides	Repaglinide and Nateglinide
	Biguanides	Metformin, Metformin XR (extended release)
	Thiazolidinediones	Rosiglitazone, Pioglitazone
	α-Glucosidase inhibitors	Acarbose, Miglitol and Yoglibos
	Dipeptidyl peptidase-4 (DPP-4) inhibitors	Sitagliptin, Saxagliptin, Vildagliptin, Linagliptin and Alogliptin
	Type-2 sodium-glucose co-transporter (SLGT2) inhibitors	Dapagliflozin, Canagliflozin, Empagliflozin and Ertugliflozin
	Dopamine agonist	Bromocriptine
	Bile acid sequestrant	Colesevelam
Injectable	Glucagon-like peptide (GLP-1) analogues	Dulaglutide, Exenatide, Semaglutide, Albiglutide, Liraglutide and Lixisenatide
	Gastric inhibitory polypeptide (GIP) and GLP-1 receptor agonist	Tirzepatide
	Amylin mimetic	Pramlintide
	Insulin	

### 2.1. Metformin and AAA

Studies on metformin treatment suggest that this drug may reduce CVD independently on its effect on improving glucose control (103–107). One of the mechanisms that have been proposed for this protective effect of metformin is through its action *via* phosphorylation of AMP-activated protein kinase (AMPK) (108), which affects several glucose-activated lipogenesis genes and GLUT4 transporter. Experimental models have shown that metformin is effective in limiting AAA progression in both DM and normoglycemic conditions. Two experimental studies showed a protective effect of metformin on AAA formation through a decrease in proinflammatory cells in the vascular wall and a reduction in proinflammatory cytokines (109, 110). A recent study in an experimental AAA model in mice showed that metformin reduced autophagy in AAA through Atg7, suggesting this molecule as a potential mediator of the protective effect of metformin in AAA (107). Besides, vascular calcification is associated with T2DM and increases the risk of cardiovascular morbidity and mortality. Pharmacological administration of metformin alleviate arterial calcification through AMPK-activated autophagy (111). In fact, it has been previously suggested that the epidemiologic evidence of the protective role of DM in AAA may be attributable to antidiabetic therapy with metformin (110). Metformin suppresses experimental AAAs and reduces enlargement rate of clinical AAAs (110, 112–115). The findings of association between metformin prescription and AAA growth rates showed that treatment with metformin in T2DM patients was associated with a reduced AAA growth in three different cohorts of approximately 1,700 patients that were analyzed by different imaging protocols (116). A systemic review and meta-analysis of 10 studies showed that patients with T2DM that were treated with metformin had slower annual AAA growth rate compared with T2DM patients without metformin. In a nationwide analysis of diabetic Veterans Affairs patients, prescription for metformin was associated with decreased AAA enlargement (112). The mechanism by which metformin may exert a protective effect on AAA may be related to its anti-inflammatory and antioxidant effect on the vasculature (109, 110, 114, 117–119) and/or to its effect on the ECM remodeling (120). Metformin treatment also reduced the frequency of rupture of AAA (121). Therefore, the translational potential use for metformin therapy is beyond the treatment of DM. Interestingly, Golledge et al. recently started a clinical trial [Metformin Aneurysm Trial (MAT)] to elucidate whether metformin reduces the risk of serious complications of AAA (122). The primary outcome of the MAT trial is the proportion of AAA events: rupture-related mortality or need for surgical repair. The secondary outcomes include AAA growth, major adverse cardiovascular events, and health-related quality of life. Another recent clinical trial (The Metformin for Abdominal Aneurysm Growth Inhibition [MAAAGI] trial started on February 2020, and had to be paused during the COVID-19 pandemia (123). In this trial, primary efficacy will be assessed by difference in AAA diameter determined by computed tomography after five years vs. at baseline. Secondary outcomes will be AAA volume and ultrasound diameter growth, rupture and elective repair AAA events, quality of life, and health economic assessment. The Limiting AAA with Metformin trial (LIMIT) started on February 2022 (124). The primary outcome will be the change in maximal orthogonal diameter of the infrarenal aorta after two years vs. at baseline and secondary outcomes will include profile of adverse cardiovascular events, all-cause mortality, change in serological markers of hepatic, hematopoietic and renal function within others. Thus, there will be very valuable data about metformin effect on AAA within the next years.

### 2.2. Thiazolidinediones and AAA

Several studies with pioglitazone have suggested a beneficial effect on CVD. These antidiabetic agents exhibit anti-inflammatory effects by reducing the levels of TNF-α (125, 126). Jones et al. demonstrated in an experimental model of Ang II-induced AAA that rosiglitazone treatment reduce inflammation and increase the aortic wall thickness by increasing collagen production. Besides, administration of rosiglitazone in mice inhibited Ang-II-mediated activation of JNK, thereby reducing the formation of aneurysms (127). It has been shown that pioglitazone decreases visceral adipose tissue, reduces cholesterol and triglycerides blood levels, increases HDL cholesterol, reduces hyperinsulinemia and IR, improves endothelial dysfunction and inflammation, risk factors for AAA development and progression (128). Thus, pioglitazone may have direct or indirect effect on AAA disease. A model of Ang II-induced AAAs in ApoE-deficient mice showed that suprarenal aortic expansion was significantly reduced by the treatment with pioglitazone compared to the control group (129). A study with rosiglitazone, another peroxisome proliferator-activated receptor-gamma agonist, showed that pretreatment or posttreatment with rosiglitazone reduced aortic expansion and rupture in the same animal model of AAA (130).

### 2.3. α-Glucosidase inhibitors and AAA

Few studies with α-glucosidase inhibitors have demonstrated protective cardiovascular effects of these hypoglycemic drugs. A nested case–control analysis using the database extracted from Taiwan's national health insurance research database showed that alpha-glucosidase inhibitors was not associated with aneurysm events (131). In this study, incidence of AAA was lower in those receiving metformin, sulfonylurea, and TZD, but not dipeptidil peptidase-4 (DPP4) inhibitors and alpha-glucosidase inhibitors. Further studies on acarbose effects on AAA disease in patients with and without DM are needed.

### 2.4. SLGT2 inhibitors and AAA

SGLT2 is a glucose transporter located in the proximal tubules of the kidneys, which is responsible for the reabsorption of the majority of the filtered glucose entering the tubules (132). Clinical and experimental data suggest that SGLT-2 inhibitors have beneficial effects in cardiovascular and metabolic diseases such as nonalcoholic steatohepatitis (133, 134), obesity (135–137), heart failure (138), and atherosclerosis; however, little is known about the effect of SGLT-2 inhibition on AAA. A recent study evaluates the effect of oral chronic treatment with empagliflozin, an SGLT-2 inhibitor, on AAA induced by Ang II infusion in mice (141). This study shows that empaglifozin treatment reduced the suprarenal aortic diameter independently of blood pressure effects. Besides, empagliflozin diminished elastin degradation, neovessel formation, macrophage infiltration and expression of CCL-2 [chemokine (C-C motif) ligand 2] and CCL-5 [chemokine (C-C motif) ligand 5], VEGF (vascular endothelial growth factor), MMP-2 and MMP-9 at the AAA lesion. In vitro studies with empaglifozin shows that this inhibitor reduces leukocyte-endothelial cell interactions and chemokine release induced by Ang II in human aortic endothelial cells (141). A more recent study on the effect of dapaglifozin on AAAs in a elastase-induced AAA experimental model (142) demonstrated that the daily treatment with dapagliflozin beginning one day prior to elastase infusion and for 14 days results in a significantly reduced aneurysmal aortic expansion. Besides, dapagliflozin reduced aortic accumulation of macrophages, CD4^+^ T cells, and B cells, attenuated medial VSMCs loss and reduced neovessel density. An experimental study demonstrated that administration of empagliflozin reverts angiotensin II-induced dissecting AAA in mice (143) possibly by reducing the expression of inflammatory chemokines, VEGF, MMP-2 and MMP9, and by reducing macrophage infiltration into the aortic wall. Empagliflozin reduces the activation of vascular p38 MAPK and NF-κB, which have been implicated in the development of AAA (143, 144).

### 2.5. GLP-1 analogs and AAA

The incretin hormone, GLP-1 is an intestinal hormone and neuronal peptide which is involved in glucose-induced insulin secretion and participates, together with GIP, in regulating glucose metabolism, energy homeostasis, control of appetite, gastrointestinal motility and trophicity. However, experimental and clinical studies have shown beneficial effects of these hormones in other pathologies and GLP-1-based therapy have been shown to prevents aneurysm formation *in vivo* (145, 146). While there is a wide knowledge of the role of GLP-1 in the context of ischemic cardiac disease, very little is known about its effect in AAA. The beneficial effect of GLP-1 in AAA might be due to its effect on the mechanisms implicated in AAA formation/progression, such as inflammation, oxidative stress, and proteolytic activity. A recent study speculates that the proteoglycan syndecan-1 (Sdc-1), whose expression is regulated by intracellular targets of the GLP-1 receptor (GLP-1R), modulates pro-inflammatory processes, and has a protective role in AAA (147). In this sense, GLP-1R activation in aortic VSMCs reverts Ang II induced reduction of Sdc-1 expression. Besides, experimental studies in both elastase and calcium chloride and Ang II-induced AAA, treatment with the GLP-1 agonist (lixisenatide and sitagliptin respectively) reduced AAA development, decreased macrophage infiltration and TNFα mRNA and MMP-9 expression in the aorta (148, 149).

### 2.6. DPP-4 inhibitors and AAA

DPP-4 inhibitors inhibits the proteolytic enzyme DPP-4 which degrades GLP-1, therefore prolonging the action of GLP-1 and resulting in enhanced glycemic control (150). Several recent studies in humans have aimed to investigate the role of DPP-4 in AAA pathogenesis. Lu et al. showed that plasma levels of DPP-4 were increased in patients with AAA and the increase was correlated with the diameter of the AAA. Besides, these authors studied the role of sitagliptin, a DPP-4 inhibitor, in a model of AAA induced by Ang II-infusion in mice. The incidence of AAA formation was significantly lower in mice treated with sitagliptin. Specifically, administered sitagliptin in Ang II-infused mice exhibited decreased expansion of the suprarenal aorta, reduced elastin lamina degradation of the aorta, and diminished vascular inflammation by macrophage infiltration (149). Noda et al. demonstrated that vildagliptin, another DPP-4 inhibitor, significantly diminished the formation of AAA and reduced expression of MMP-2, MMP-9, and IL-6 an experimental model of calcium chloride-induced AAA (151). An experimental study with teneligtip showed that AAA formation was significantly reduced in the treated group compared with the control group. Teneligtip also retarded AAA growth, reduced elastin degradation and reduced macrophage infiltration (152). Similarly, a rat model of AAA showed that alogliptin attenuates aneurysm formation and aortic dilatation ratio *via* an antioxidative effect (reduction of ROS formation), and reduced MMP2 and MMP-9 expression levels in the aortic tissue (153). Two different studies with the non-selective DPP-4 inhibitor MK0626 in murine models of AAA demonstrated a reduced AAA formation in the treated group compared to the control group that was related with changes in the ECM (154, 155).

A decreased infiltration of macrophages in the aortic wall may be, at least in part, responsible of the protective effect of GLP-1 analogs on AAA formation and progression (156). Studies in mice have shown that TNFα expression levels are increased in aneurysm tissue compared with healthy aortic tissues. Pharmacological and genetic inhibition of TNFα expression decreases AAA and, therefore, the protective effect of GLP-1R agonists limiting AAA development might be due to its effect on TNFα expression (157). Similarly, administration of GLP-1 agonist or DPP-4 inhibitors reduces the expression of MMP-2 and MMP-9 in the aorta. This effect may account for the protective effect of these drugs on preserving the ECM and therefore, on reducing the progression of AAA (158).

## 3. Conclusion

Although some DM-related factors might protect against AAA formation and progression (e.g., hyperglycemia-modulation of ECM), most features of DM disease are part of the risk factors which are associated with CVD and, most likely, contribute in some degree to the development of AAA. Most published reviews on the association of DM and AAA do not emphasize differences between T1DM and T2DM. In fact, experimental investigation on the study of T2DM and AAA in T2DM mouse models are lacking. Analyzing the differences between the effect of T1DM and T2DM on the formation and progression of AAA may help elucidating the question of whether DM is protective for AAA. Besides, the relationship between AAA and insulin resistance and/or the pre-diabetic state is poorly understood and needs further investigation. Furthermore, the ability of adipose tissue and its secreted adipokines to modulate the vascular environment may require more attention since increased adiposity is one of the main features in T2DM. At the same time, some of the clinical studies linking a protective role of DM in the incidence and progression of AAA do not specify whether those patients are receiving antidiabetic treatments. In addition, controversial results between non-postoperative and postoperative data reveal non-conclusive evidence of the protective effect of DM *per se* in AAA. The main challenge in these studies in humans is the availability of patients with AAA with no previous drug prescription.

It is being established that the protective effect against AAA is, at least in part, dependent on the pharmacological treatments used for DM. In this regard, recent available literature shows clear protective effects of antidiabetic drugs (metformin, SGLT2-inhibitors, incretin-based therapies, among others) on the incidence and progression of AAA. Although some of these drugs have recently been introduced and cannot explain old observations, other antidiabetic agents such as metformin have been used before and may explain, at least in part, the AAA progression in patients with DM. In addition, new data from specific clinical trials will be available within the next years that will help elucidating the mechanisms by which these drugs protect against AAA.
